# Whole genome sequencing and phenotypic characterization of microbial isolates associated with two Floridian fig species

**DOI:** 10.17912/micropub.biology.002288

**Published:** 2026-08-31

**Authors:** Isabella Hall, Jenna Naumann, Shelby G. Rinderer, Ally J. Whitson, Pamela J.B. Brown, Justin Van Goor

**Affiliations:** 1 Biological Sciences, University of Missouri-Columbia

## Abstract

Fig-associated microbes are increasingly recognized as important contributors to the ecology of fig ( *Ficus* ) communities, yet few cultured isolates are available for functional investigation. Students enrolled in a course-based undergraduate research experience (CURE) phenotypically characterized and generated high-quality genome assemblies for 22 bacterial isolates recovered from *Ficus aurea* and *Ficus citrifolia* in southern Florida, USA. These isolates represent six bacterial species spanning four genera and provide a genomic resource for future investigations of microbial interactions within fig communities.

**Figure 1. Phylogenetic relationships and phenotypic characteristics of fig-associated bacterial isolates:** **(A)** Bayesian phylogeny based on a concatenated dataset of *16S* rRNA, *23S* rRNA, *rpoB* , *gyrB* , and *recA* gene sequences of bacterial isolates recovered from fig syconia. All nodes have a posterior probability value of >.95. Branch colors denote the major taxonomic groups represented in panel B. **(B)** Representative phenotypic characteristics of each genus. Phase-contrast micrographs of representative isolates are shown. Gram stain results were consistent with taxonomic classification. Swimming motility was assessed by wet-mount microscopy and corresponded with the presence or absence of predicted flagellar biosynthesis pathways identified using BlastKOALA (Kanehisa M., *et al* ., 2016). A diagonal line indicates the absence of swimming motility. Antagonistic activity against *Aspergillus niger* and *Saccharomyces cerevisiae* was evaluated using mycelial growth inhibition and yeast growth inhibition assays, respectively. Open icons indicate no inhibition, dashed lines indicate variable inhibition among isolates within a genus, and solid lines indicate inhibition by all isolates within the genus. Evidence of β-lactamase production was observed in all *Kosakonia* isolates using disk diffusion assays with ampicillin and ampicillin-sulbactam disks. Siderophore biosynthetic gene clusters were identified in *Pantoea* and *Klebsiella–Raoultella* complex isolates using antiSMASH (Blin K., *et al* ., 2025). Strain-specific phenotypic results are reported in Table 1. Table 1: Whole genome sequencing statistics and strain-specific phenotypes for fig-associated bacterial isolates

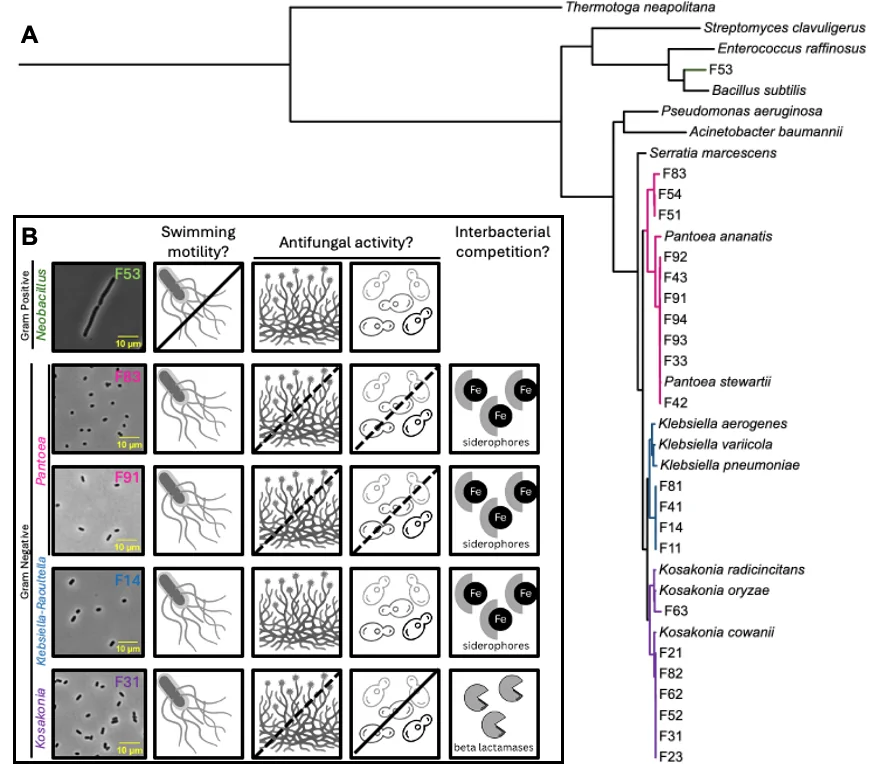



**Table:** 

Strain	Genome Size (bp)	% Completeness	rNG50 (bp)	# of Contigs	Assembly Accession Number	Assembled Coverage	# of annotated genes	Closest Named Relative (Accession Number)	Estimated %ID	β-lactamase Production?	Swimming Motility?	Aspergillus Inhibition?	Yeast Inhibition?
F31	4786879	99.42	48712	1	GCA_056712235.1	68x	4426	Kosakonia cowanii strain LT-1 (NZ_CP107077.1)	94.1	Yes	Yes	No	Yes
F62	4786543	99.42	53194	1	GCA_056712205.1	164x	4427	Kosakonia cowanii strain LT-1 (NZ_CP107077.1)	94.1	Yes	Yes	No	Yes
F23	4786881	99.42	51333	1	GCA_056712085.1	75x	4427	Kosakonia cowanii strain LT-1 (NZ_CP107077.1)	94.1	ND	ND	Yes	Yes
F21	4786879	99.42	55044	1	GCA_056712065.1	137x	4426	Kosakonia cowanii strain LT-1 (NZ_CP107077.1)	94.1	Yes	Yes	Yes	Yes
F82	4783961	99.42	50699	1	GCA_056712325.1	141x	4427	Kosakonia cowanii strain LT-1 (NZ_CP107077.1)	94.1	ND	Yes	Yes	Yes
F52	4786881	99.42	58571	1	GCA_056712445.1	136x	4426	Kosakonia cowanii strain LT-1 (NZ_CP107077.1)	94.1	Yes	Yes	Yes	Yes
F63	5820362	99.6	54487	7	GCA_056712165.1	121x	5921	Kosakonia oryzae strain Ola 51 (NZ_CP014007.2)	96.9	No	Yes	No	Yes
F53	6910346	99.39	25526	1	GCA_056712425.1	46x	6567	Neobacillus niacini strain BE6 (NZ_CP183883.1)	79.6	No	No	No	No
F51	6383044	100	61637	11	GCA_056712475.1	118x	6461	Pantoea phytobeneficialis strain MSR2 (NZ_CP024636.1)	93	No	No	Yes	No
F54	6017837	100	55253	8	GCA_056712385.1	86x	6080	Pantoea phytobeneficialis strain MSR2 (NZ_CP024636.1)	93	No	Yes	No	NG
F83	6497431	100	54701	11	GCA_056712285.1	202x	6723	Pantoea phytobeneficialis strain MSR2 (NZ_CP024636.1)	93	ND	ND	Yes	Yes
F94	4995305	99.34	46821	3	GCA_056712105.1	67x	4705	Pantoea stewartii strain ZJ-FGZX1 (NZ_CP049115.1)	98.9	ND	ND	Yes	Yes
F42	5067589	99.34	56358	3	GCA_056712405.1	83x	4755	Pantoea stewartii strain ZJ-FGZX1 (NZ_CP049115.1)	98.9	ND	Yes	Yes	Yes
F33	5056945	99.34	50871	3	GCA_056712225.1	94x	4732	Pantoea stewartii strain ZJ-FGZX1 (NZ_CP049115.1)	98.9	ND	Yes	Yes	NG
F91	5025771	99.34	50420	3	GCA_056712175.1	116x	4761	Pantoea stewartii strain ZJ-FGZX1 (NZ_CP049115.1)	98.9	ND	Yes	Yes	Yes
F93	4997194	99.34	53433	3	GCA_056712035.1	155x	4712	Pantoea stewartii strain ZJ-FGZX1 (NZ_CP049115.1)	98.9	ND	ND	No	Yes
F92	5059300	99.34	46466	3	GCA_056712115.1	65x	4738	Pantoea stewartii strain ZJ-FGZX1 (NZ_CP049115.1)	99	Yes	No	Yes	Yes
F43	5059293	99.34	60952	3	GCA_056712345.1	233x	4735	Pantoea stewartii strain ZJ-FGZX1 (NZ_CP049115.1)	99	No	Yes	Yes	Yes
F41	6104246	99.69	60491	4	GCA_056712365.1	89x	5945	Raoultella terrigena strain JH01 (NZ_CP050508.1)	93.8	ND	ND	No	No
F81	5931355	99.63	49078	82	GCA_056712305.1	72x	5692	Raoultella terrigena strain JH01 (NZ_CP050508.1)	93.8	No	Yes	No	No
F11	6104246	99.69	54795	4	GCA_056712265.1	134x	5944	Raoultella terrigena strain JH01 (NZ_CP050508.1)	93.8	ND	ND	ND	ND
F14	6268432	99.69	54018	7	GCA_056712145.1	130x	6151	Raoultella terrigena strain JH01 (NZ_CP050508.1)	93.8	ND	Yes	No	No

## Description

Figs ( *Ficus* spp.) harbor diverse communities of animals, fungi, and microbes that interact within the enclosed environment of the fig syconium itself. Although the fig–fig wasp mutualism has served as a model for studying coevolution for decades (Cook and Rasplus 2003), comparatively little is known about the microbial members of these communities and their ecological roles. Recent work has demonstrated that the fig community harbors a diverse assemblage of bacterial associates (Woodruff et al. 2026), yet our understanding of bacterial functional ecologies within the fig environment remains muddied. Interestingly, other recent research suggests that bacterial associates may mitigate fungal pathogens within figs (Van Goor et al. 2026), highlighting the need for cultured isolates and genomic resources to investigate these interactions. This raises an important question: which bacteria are implicated in fungal mitigation, and why?

To support future studies of fig-associated microbial communities, bacterial isolates were recovered from recently pollinated syconia of two fig species native to southern Florida, USA ( *Ficus aurea* and *Ficus citrifolia* ). The isolates served as the basis for a course-based undergraduate research experience (CURE) in the University of Missouri Microbiology Laboratory course (BIO_SC 3760). Course-based undergraduate research experiences are high-impact educational practices that improve student learning and retention while simultaneously generating valuable biological datasets (Martinez-Vaz and Bell 2025; Ksiazek Mikenas 2026).

The interior of fig syconia were aseptically sampled and plated on lysogeny broth (LB) agar. Individual colonies were purified by repeated streaking, and each strain was maintained as a frozen stock with 25% glycerol at −80 °C. Each isolate is available by request from the Brown lab. During the semester, each student characterized one of the 22 bacterial isolates through phenotypic analyses, including Gram staining, swimming motility, β-lactamase screening, and antagonistic activity against *Aspergillus niger* and *Saccharomyces cerevisiae* .

For whole-genome sequencing, isolates were cultured on LB agar for 2 days at 30 °C before a single colony was inoculated into 6 mL LB medium and grown with shaking for 2 days at 30 °C. Cells were pelleted (13,000 × *g* ), washed with phosphate-buffered saline (PBS), resuspended in 0.5 mL DNA/RNA Shield (Zymo Research), and submitted to Plasmidsaurus (Eugene, OR, USA) for Oxford Nanopore sequencing (Purushothaman et al. 2026). Genome assembly and annotation were performed using the Plasmidsaurus bacterial assembly pipeline, which incorporates Filtlong (Wick 2026), Flye (Kolmogorov et al. 2019), Autocycler (Wick et al. 2025), Dnaapler (Bouras et al. 2024), and Plassembler (Bouras et al. 2023) for read filtering, genome assembly, consensus generation, genome reorientation, and plasmid assembly, respectively. Whole genome sequencing statistics for fig-associated bacterial isolates are shown in Table 1 and illustrate excellent coverage, completeness, and rNG50 values. Genome assemblies and raw sequence data have been deposited under BioProject PRJNA1419296 with associated BioSample accessions SAMN55952996–SAMN55953017 and Sequence Read Archive accessions SRX33714672–SRX33714693.

Genome assemblies were used for taxonomic classification, phylogenetic analysis, and functional annotation. As part of the Plasmidsaurus pipeline Mash (Ondov et al 2016) was used to identify the closest named relative available in RefSeq (O’Leary et al 2016) for the largest contig in each genome assembly (Table 1). The estimated percent identity (Estimated %ID, Table 1) is estimated from the number of *k* -mers that matched the reference genome for the closed named relative. To validate and refine the taxonomic classification of the isolates, a multiple sequence concatenated alignment of 16S rRNA, 23S rRNA, RNA polymerase beta subunit ( *rpoB* ), DNA gyrase subunit B ( *gyrB* ), and recombinationA ( *recA* ) gene sequences were generated using MUSCLE (Edgar 2004) implemented in Geneious Prime V2025.2.2 (http://www.geneious.com). Bayesian phylogenetic inference was performed with MrBayes (Huelsenbeck and Ronquist 2001) using the GTR+G+I substitution model and 5,000,000 generations. Trees were edited in FigTree (Rambaut 2018) and visualized with *Thermotoga neapolitana* as the outgroup using Interactive Tree of Life (iTOL; Letunic and Bork 2024). Functional annotation was performed using BlastKOALA (Kanehisa et al. 2016), and candidate antimicrobial and secondary metabolite biosynthetic gene clusters were identified using antiSMASH (Blin et al. 2025).

Whole-genome sequencing and taxonomic analyses identified 22 isolates representing six bacterial species spanning four genera: *Klebsiella-Raoultella* complex, *Kosakonia* , *Neobacillus* , and *Pantoea* ( Figure 1A ). Representative phenotypes are shown and summarized in Figure 1B and strain-specific phenotypic profiles are indicated in Table 1 (ND = not determined). The *Klebsiella-Raoultella* complex, *Kosakonia* , and *Pantoea* isolates were motile, Gram-negative rods, although substantial variation was observed in antagonistic activity against fungi ( Figure 1B, Table 1). In contrast, the sole Gram-positive bacterium in our dataset (presumed *Neobacillus* ) displayed no apparent motility, no detectable antifungal inhibition behaviors, and no apparent microbial competition abilities ( Figure 1B, Table 1).

As expected in all communities, this analysis of the microbial communities of fig species identifies associates with varying ecologies. When viewing through the scope of potential fungal mitigation, our phenotypic analyses (Table 1, Figure 1B ) strongly suggest that isolated members of the *Klebsiella-Raoultella complex* and *Neobacillus* function as commensals within the internal fig environment (Mathis and Bronstein 2020). However, members of the *Klebsiella–Raoultella* complex and the genus *Neobacillus* have been described as plant growth promoters in other agricultural systems (Singh et al. 2015, Kumar et al. 2021, Tsotetsi et al. 2022), particularly under environmental/abiotic stressors. It is possible that they are commensals, or that they interact with figs in ways that our current experiments have not explored.

Intriguingly, *Kosakonia* and *Pantoea* isolates consistently showed antifungal activities *and* evidence of antimicrobial molecular defenses, both of which suggest a protective mutualism between these microbes and the figs they inhabit (Table 1, Figure 1B ). Indeed, isolates of *Kosakonia* consistently inhibited the growth of *S. cerevisiae* and most isolates also inhibited the growth of both *A. niger* . Some *Kosakonia* isolates exhibited β-lactamase activity, and genes encoding predicted β-lactamases were identified in their genomes. Similarly, most of the *Pantoea* isolates inhibited the growth of *S. cerevisiae* and *A. niger* and their genomes contain predicted siderophore biosynthetic gene clusters (Table 1, Figure 1B ). These data suggest that *Kosakonia* and *Pantoea* isolates may have some beneficial properties in fungal mitigation or interbacterial competition within the fig microenvironment. While *Kosakonia* and *Pantoea* have both been previously implicated in fungal mitigation for other plants (Jiang et al. 2019, Thanwisai et al. 2024), this is the first experimental evidence of such activities in fig associates. These findings may inform future management of fungal diseases for the cultivated fig, such as Fig Endosepsis (Michailides and Morgan 1998, Van Goor et al. 2026). Further, while microbially produced molecules such as siderophores are known to benefit associates through nutrient acquisition and competitive abilities (Kramer et al. 2019), production of siderophores by *Kosakonia* has also been linked to fungal mitigation abilities (Lambrese et al. 2018), warranting future examination within the context of the fig community. Together, these genome assemblies and accompanying phenotypic analyses provide a valuable resource for future investigations into the ecological roles of bacteria within fig-associated microbial communities.

While the data presented here suggest a promising connection between the presence of some fig microbes and the mitigation/limitation of fungal growth, it also presents a curious conundrum: if anti-fungal bacteria are present within the fig environment, why do we see figs infested with fungus? Local infestation rate of *Fusarium* fungus within the Floridian fig species examined here sometimes exceeded 95% of the figs sampled; how could this be? Future research efforts will prioritize the examination of multiple microbial taxa working in concert, and the potential role of alternative eukaryotic fig associates (notably nematodes) in the mitigation of pathogenic fungus. Finally, it appears as if our previous culturing efforts largely selected for Gram-negative bacteria, but more recent culture-independent metabarcode efforts suggest the presence of a large diversity of Gram-positive bacteria that were present within the fig environment as well. Future iterations of the BIO_SC 3760 CURE course seek to utilize alternative media in an effort to culture a wider diversity of microbial associates to further understand their functional ecological roles within the fig environment.

## Methods

**Bacterial strains and growth conditions**

All bacterial strains used in this study were grown in LB Miller broth (10 g tryptone, 5 g yeast extract, and 10 g NaCl L⁻¹) or agar at 30°C with shaking unless otherwise indicated.

**Phase contrast microscopy**

To prepare for microscopy, strains were grown to exponential phase in LB medium and 0.5 mL of cells were spotted onto 1.75% LB low-melt agarose pads. Cells were imaged using an inverted Nikon Eclipse TiE equipped with a 63X 1.4 NA Plan Apochromat oil-immersion phase-contrast objective using a Rolera em-c ^2^ 1K EMCCD and Nikon Elements Imaging Software.

**Gram staining**

A thin smear was prepared by suspending a fresh bacterial colony in 5 µL sterile water on a glass microscope slide. Smears were air dried for 10–15 min, heat fixed, and Gram stained using a Remel™ Gram Stain Kit (Thermo Fisher Scientific) according to the manufacturer's instructions. Gram-stained cells were observed and imaged using a Leica ICC50 W microscope equipped with a 40× brightfield objective.

**Wet mount microscopy**

Bacterial isolates were grown overnight in 1 mL LB medium with shaking at 30 °C. A 5 µL aliquot of culture was placed on a glass microscope slide, covered with a coverslip, and examined using a Leica ICC50 W microscope equipped with a 100× oil immersion phase-contrast objective. Phase-contrast videos were recorded to document the presence or absence of swimming motility.

**β-Lactamase screening**

Bacterial isolates were screened for β-lactamase production using a disk diffusion assay on Mueller-Hinton agar. Bacterial lawns were prepared from overnight cultures, and commercial ampicillin and ampicillin-sulbactam disks were placed onto the agar surface. Plates were incubated at 30 °C for 2 days, after which zones of inhibition were measured in millimeters. Increased susceptibility to ampicillin in the presence of sulbactam was interpreted as evidence of β-lactamase production.

**Antagonistic activity against fungi**

*Yeast growth inhibition assay (Saccharomyces cerevisiae).* Commercial active dry baker's yeast ( *Saccharomyces cerevisiae* ) was prepared by hydrating 1 g of yeast in 10 mL sterile water for 10 min and vortexing to obtain a homogeneous suspension. The suspension was serially diluted from 10 ^-2^ to 10 ^-6^ in sterile water. Bacterial isolates were spread onto yeast peptone dextrose (YPD) agar using sterile cotton swabs to produce confluent lawns and allowed to dry for approximately 15 min. Five-microliter aliquots of each yeast dilution were spotted in technical duplicate onto bacterial lawns and uninoculated control plates. After the inoculum had dried, plates were sealed with parafilm and incubated at 30 °C. After 2 days of incubation, yeast growth on bacterial lawns was compared with growth on control plates lacking bacteria. Growth inhibition was determined by comparing the maximum dilution supporting visible yeast growth on bacterial lawns with the maximum dilution supporting visible growth on control plates and was reported as the corresponding log-fold reduction in yeast growth.

*Mycelial growth inhibition assay (Aspergillus niger).* *Aspergillus niger* was obtained as a live culture from Carolina Biological Supply (Burlington, NC, USA) and maintained on potato dextrose agar (PDA). Bacterial isolates were spread onto PDA using sterile cotton swabs to produce confluent lawns and allowed to dry for approximately 15 min. A 1-cm² plug of actively growing *A. niger* mycelium was excised from the stock culture and transferred to the center of each bacterial lawn. Control plates consisted of fungal plugs transferred to PDA without bacterial lawns. Plates were sealed with parafilm, incubated in an inverted orientation at 30 °C, and monitored for fungal growth. Mycelial diameter was measured on days 2, 5, and 7. Fungal mat area was calculated from colony diameter, assuming circular colony morphology, and used to quantify fungal growth.

## References

[R1] Blin K, Shaw S, Vader L, Szenei J, Reitz ZL, Augustijn HE, Cediel-Becerra JDD, de Crécy-Lagard V, Koetsier RA, Williams SE, Cruz-Morales P, Wongwas S, Segurado Luchsinger AE, Biermann F, Korenskaia A, Zdouc MM, Meijer D, Terlouw BR, van der Hooft JJJ, Ziemert N, Helfrich EJN, Masschelein J, Corre C, Chevrette MG, van Wezel GP, Medema MH, Weber T (2025). antiSMASH 8.0: extended gene cluster detection capabilities and analyses of chemistry, enzymology, and regulation.. Nucleic Acids Res.

[R2] Bouras George, Grigson Susanna R., Papudeshi Bhavya, Mallawaarachchi Vijini, Roach Michael J. (2024). Dnaapler: A tool to reorient circular microbial
genomes. Journal of Open Source Software.

[R3] Bouras G, Sheppard AE, Mallawaarachchi V, Vreugde S (2023). Plassembler: an automated bacterial plasmid assembly tool.. Bioinformatics.

[R4] Cook James M., Rasplus Jean-Yves (2003). Mutualists with attitude: coevolving fig wasps and figs. Trends in Ecology & Evolution.

[R5] Edgar RC (2004). MUSCLE: multiple sequence alignment with high accuracy and high throughput.. Nucleic Acids Res.

[R6] Huelsenbeck JP, Ronquist F (2001). MRBAYES: Bayesian inference of phylogenetic trees.. Bioinformatics.

[R7] Jiang Lingmin, Jeong Jae Chul, Lee Jung-Sook, Park Jeong Mee, Yang Jung-Wook, Lee Myoung Hui, Choi Seung Hee, Kim Cha Young, Kim Dae-Hyuk, Kim Suk Weon, Lee Jiyoung (2019). Potential of Pantoea dispersa as an effective biocontrol agent for black rot in sweet potato. Scientific Reports.

[R8] Kanehisa M, Sato Y, Morishima K (2015). BlastKOALA and GhostKOALA: KEGG Tools for Functional Characterization of Genome and Metagenome Sequences.. J Mol Biol.

[R9] Kolmogorov M, Yuan J, Lin Y, Pevzner PA (2019). Assembly of long, error-prone reads using repeat graphs.. Nat Biotechnol.

[R10] Kramer Jos, Özkaya Özhan, Kümmerli Rolf (2019). Bacterial siderophores in community and host interactions. Nature Reviews Microbiology.

[R11] Ksiazek-Mikenas Kelly (2026). A course-based undergraduate research experience (CURE) improves both student learning and restoration outcomes. Journal of Environmental Studies and Sciences.

[R12] Kumar Govind, Lal Shatrohan, Maurya Shailendra K., Bhattacherjee A. K., Chaudhary Parul, Gangola Saurabh, Rajan Shailendra (2021). Exploration of Klebsiella pneumoniae M6 for paclobutrazol degradation, plant growth attributes, and biocontrol action under subtropical ecosystem. PLOS ONE.

[R13] Lambrese Yesica, Guiñez María, Calvente Viviana, Sansone Gabriela, Cerutti Soledad, Raba Julio, Sanz María Isabel (2018). Production of siderophores by the bacterium Kosakonia radicincitans and its application to control of phytopathogenic fungi. Bioresource Technology Reports.

[R14] Letunic I, Bork P (2024). Interactive Tree of Life (iTOL) v6: recent updates to the phylogenetic tree display and annotation tool.. Nucleic Acids Res.

[R15] Martinez-Vaz Betsy M., Bell Ellis (2025). Molecular Life Science CUREs: Design, Implementation, and Assessment. ACS Symposium Series.

[R16] Mathis Kaitlyn A., Bronstein Judith L. (2020). Our Current Understanding of Commensalism. Annual Review of Ecology, Evolution, and Systematics.

[R17] Michailides Themis J., Morgan David P. (1998). Spread of Endosepsis in Calimyrna Fig Orchards. Phytopathology®.

[R18] O'Leary NA, Wright MW, Brister JR, Ciufo S, Haddad D, McVeigh R, Rajput B, Robbertse B, Smith-White B, Ako-Adjei D, Astashyn A, Badretdin A, Bao Y, Blinkova O, Brover V, Chetvernin V, Choi J, Cox E, Ermolaeva O, Farrell CM, Goldfarb T, Gupta T, Haft D, Hatcher E, Hlavina W, Joardar VS, Kodali VK, Li W, Maglott D, Masterson P, McGarvey KM, Murphy MR, O'Neill K, Pujar S, Rangwala SH, Rausch D, Riddick LD, Schoch C, Shkeda A, Storz SS, Sun H, Thibaud-Nissen F, Tolstoy I, Tully RE, Vatsan AR, Wallin C, Webb D, Wu W, Landrum MJ, Kimchi A, Tatusova T, DiCuccio M, Kitts P, Murphy TD, Pruitt KD (2015). Reference sequence (RefSeq) database at NCBI: current status, taxonomic expansion, and functional annotation.. Nucleic Acids Res.

[R19] Ondov BD, Treangen TJ, Melsted P, Mallonee AB, Bergman NH, Koren S, Phillippy AM (2016). Mash: fast genome and metagenome distance estimation using MinHash.. Genome Biol.

[R20] Purushothaman S, Roloff T, Egli A, Seth-Smith HM (2026). Benchmarking Illumina and Oxford Nanopore Technologies (ONT) sequencing platforms for whole genome sequencing of bacterial genomes and use in clinical microbiology.. BMC Med Genomics.

[R21] Rambaut A. 2018. FigTree. Version 1.4.4 [software]. Available from: https://tree.bio.ed.ac.uk/software/figtree/.

[R22] Singh Rajnish Prakash, Jha Prameela, Jha Prabhat Nath (2015). The plant-growth-promoting bacterium Klebsiella sp. SBP-8 confers induced systemic tolerance in wheat (Triticum aestivum) under salt stress. Journal of Plant Physiology.

[R23] Thanwisai Lalita, Siripornadulsil Wilailak, Siripornadulsil Surasak (2024). Kosakonia oryziphila NP19 bacterium acts as a plant growth promoter and biopesticide to suppress blast disease in KDML105 rice. Scientific Reports.

[R24] Tsotetsi T, Nephali L, Malebe M, Tugizimana F (2022). Bacillus for Plant Growth Promotion and Stress Resilience: What Have We Learned?. Plants (Basel).

[R25] Van Goor Justin, Killmade Maya, Gómez Zúñiga Adalberto, Herre Edward Allen, Piatscheck Finn, Houston Derek D., Michailides Themis, Palomares‐Rius Juan Emilio, Bever James D., Haag Eric S. (2026). Species introduction woes: How human‐mediated crop transport can influence community dynamics through mutualist displacement. Journal of Applied Ecology.

[R26] Wick R. 2026. Filtlong [software]. GitHub. Available from: https://github.com/rrwick/Filtlong.

[R27] Wick RR, Howden BP, Stinear TP (2025). Autocycler: long-read consensus assembly for bacterial genomes.. Bioinformatics.

[R28] Woodruff GC, Moser KA, Wang J (2026). The bacteria of a fig community.. Microbiol Spectr.

